# Diagnosis of Serosal Invasion in Gastric Adenocarcinoma by Dual-Energy CT Radiomics: Focusing on Localized Gastric Wall and Peritumoral Radiomics Features

**DOI:** 10.3389/fonc.2022.848425

**Published:** 2022-03-21

**Authors:** Li Yang, Junyi Sun, Xianbo Yu, Yang Li, Min Li, Jing Liu, Xiangming Wang, Gaofeng Shi

**Affiliations:** ^1^ Department of Computed Tomography and Magnetic Resonance, Fourth Hospital of Hebei Medical University, Shijiazhuang, China; ^2^ CT Collaboration, Siemens Healthineers Ltd., Beijing, China

**Keywords:** stomach neoplasms, serosal invasion, dual-energy CT, iodine map, radiomics

## Abstract

**Objectives:**

To build a radiomics model and combined model based on dual-energy CT (DECT) for diagnosing serosal invasion in gastric adenocarcinoma.

**Materials and methods:**

231 gastric adenocarcinoma patients were enrolled and randomly divided into a training (*n* = 132), testing (*n* = 58), and independent validation (*n* = 41) cohort. Radiomics features were extracted from the rectangular ROI of the 120-kV equivalent mixed images and iodine map (IM) images in the venous phase of DECT, which was manually delineated perpendicularly to the gastric wall in the deepest location of tumor infiltration, including the peritumoral adipose tissue within 5 mm outside the serosa. The random forest algorithm was used for radiomics model construction. Traditional features were collected by two radiologists. Univariate and multivariate logistic regression was used to construct the clinical model and combined model. The diagnostic efficacy of the models was evaluated using ROC curve analysis and compared using the Delong’s test. The calibration curves were used to evaluate the calibration performance of the combined model.

**Results:**

Both the radiomics model and combined model showed high efficacy in diagnosing serosal invasion in the training, testing and independent validation cohort, with AUC of 0.90, 0.90, and 0.85 for radiomics model; 0.93, 0.93, and 0.89 for combined model. The combined model outperformed the clinical model (AUC: 0.76, 0.76 and 0.81).

**Conclusion:**

The radiomics model and combined model constructed based on tumoral and peritumoral radiomics features derived from DECT showed high diagnostic efficacy for serosal invasion in gastric adenocarcinoma.

## Introduction

In China, the incidence and mortality of gastric cancer remain high, and the majority of patients are in the advanced stage (1). Gastric serosa has a defensive function, preventing tumor cells from spreading to the surroundings. The risk of peritoneal metastasis (PM) increases after serosal invasion in gastric cancer patients (2–5). According to the 2016 clinical practice guidelines for gastric cancer established by the National Comprehensive Cancer Network (NCCN) (6), neoadjuvant chemotherapy (NAC) is recommended for patients with T4 advanced gastric cancer to achieve lower staging, improve R0 resection rate, and improve prognosis. Both European Society for Medical Oncology (ESMO) (7) and National Comprehensive Cancer Network (NCCN) guidelines (6) recommended that laparoscopy exploration should be applied to patients with advanced gastric cancer to detect occult PM. However, laparoscopy is an invasive diagnostic procedure and cannot directly determine serosal invasion. Currently, postoperative pathology is the gold standard for the diagnosis of serosal invasion, but it has a certain hysteresis. Therefore, if the serosal status can be accurately determined preoperatively, most inappropriate surgeries will be avoided.

MDCT is a first-line imaging modality for the preoperative evaluation of gastric cancer (8, 9). CT findings (10–12), (such as serosal nodule, enhancement of the serosa, perigastric fatty infiltration, and perigastric vascular invasion) are common indicators for serosal invasion. However, inflammatory peritumoral reaction and lack of perigastric adipose tissue can affect the determination of serosal status. Additionally, in cases of serosal microinvasion, it is difficult to observe the typical features of the serosal surface. CT accuracy for diagnosis of serosal invasion was reported to be 55.9%–90.8% (10–13). Thus, more objective and quantitative parameters are required for detecting serosal invasion more accurately.

Dual-energy CT (DECT) can provide more information without increasing the radiation dose (14, 15). It has been reported that the quantification of iodine concentration (IC) with DECT in peritumoral adipose tissue provides an accurate method for detecting serosal invasion in gastric cancer (16).

Radiomics is an emerging tool to quantitatively assess lesion characteristics from texture analysis techniques (17). A recent study showed that a radiomics model had a relatively high diagnostic efficacy for serosal invasion compared with a traditional model based on conventional CT signs (18). Another radiomics study showed that iodine map (IM) derived from DECT increased the diagnostic value of restaging in advanced gastric cancer patients after NAC (19). The volumes of interest (VOIs) were delineated in three dimensions without the peritumoral tissue in the above two studies. Numerous studies have demonstrated that the peritumoral radiomics features may provide important clues for predicting tumor aggressiveness (20, 21).

This study is aimed to construct a radiomics model and combined model based on tumoral and peritumoral radiomics features derived from DECT using a time-saving delineation method and evaluate their diagnostic efficacy for predicting serosal invasion in gastric adenocarcinoma.

## Materials and Methods

### Characteristics of Patients

We retrospectively reviewed the data of gastric cancer patients in our institution between April 2015 and December 2017. This study has been approved by the Ethics Committee of The Fourth Hospital of Hebei Medical University. Inclusion and exclusion criteria were as follows:

Inclusion criteria: (1) Gastric cancer was confirmed by gastroscopic biopsy and the patients had not received any antitumor therapy (e.g., radiotherapy, chemotherapy, and targeted therapy) before surgery. (2) Dual-energy abdominal enhancement scan was performed within 2 weeks before surgery, and the complete imaging data were available. (3) Gastric adenocarcinoma was confirmed by postoperative pathology with clear staging.

Exclusion criteria: (1) Inadequate preparation for CT examination (e.g., poor gastric filling and excessive food retention). (2) The presence of breathing or sclerotic artifacts in the image. (3) The lesion was too small to be detected by CT. (4) There was too little peritumoral adipose tissue to meet the requirements of the ROI delineation.

**Figure 1:**
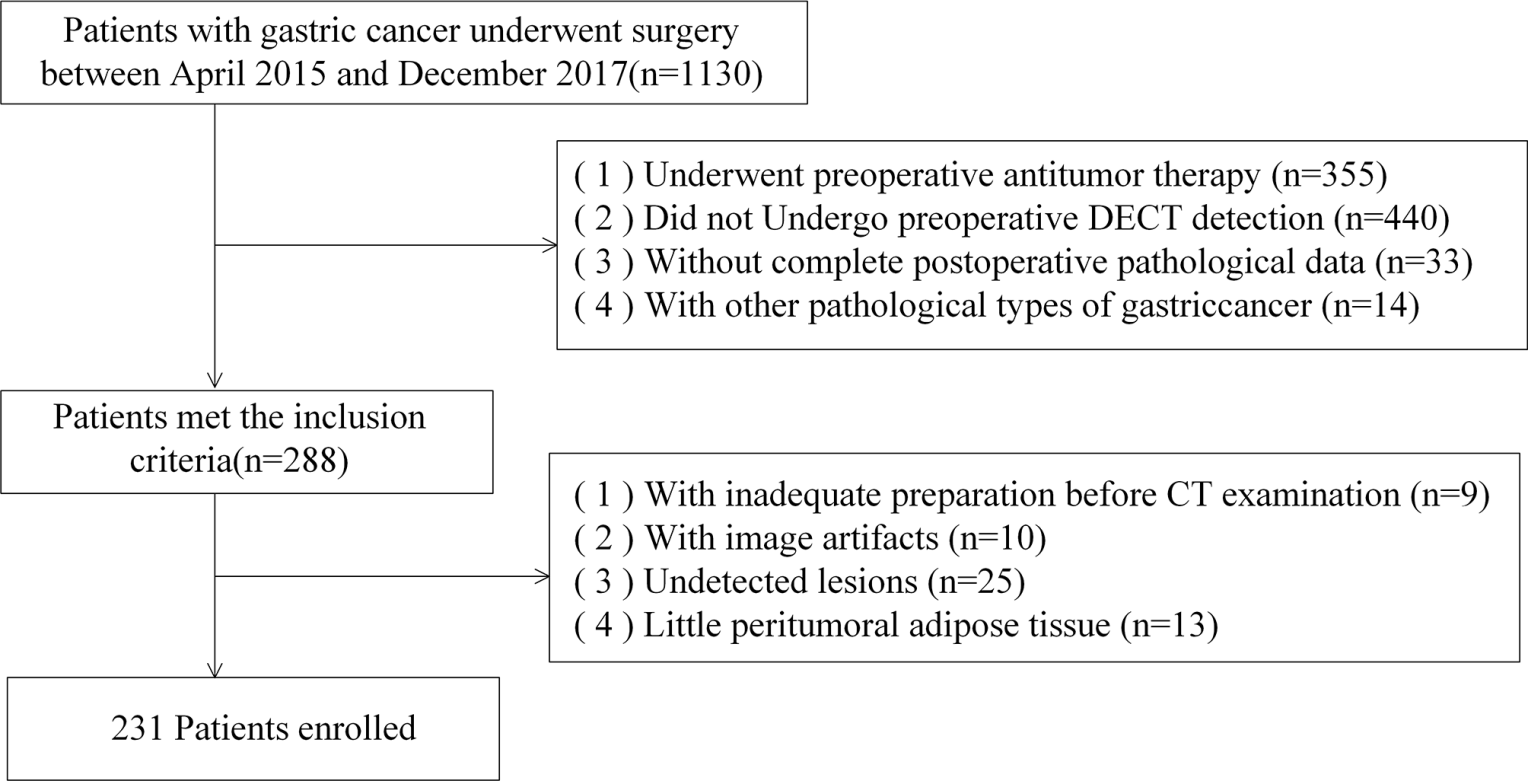
Flowchart of patient enrollment.

231 patients were eligible for enrollment (**Figure 1**). The patients were randomly divided into the developing and independent validation set at the ratio of 8:2, respectively. Moreover, the patients in the developing set were randomly divided into the training set and testing set at the ratio of 7:3.

### Imaging Protocol and Postprocessing

#### Patient Preparation

After fasting for 6–8 hours, all patients received intramuscular injection of 10 mg of 654-2 (anisodamine) 10 min before the examination, and orally administered 6 g of aerogenic powder with a small volume of tap water to fill the gastric cavity.

#### Examination Method and Scanning Parameters

The scan was performed using a dual-source CT scanner (SOMATOM Definition Flash; Siemens Healthcare, Germany). The scan range was from 5 cm above the diaphragm to the level of the lower margin of the symphysis pubis. The patients maintained a supine position during the examination. The plain scan parameters were as follows: tube voltage:120 kVp; tube current: 210 mAs; collimation width 32 × 1.2 mm, and pitch: 0.9. Non-ionic contrast agent (Iohexol, 300 mg/dL; GE Healthcare, USA) was injected intravenously through the elbow vein at a flow rate of 3 ml/s (2 mL/kg body weight). Two phase-enhanced with dual-energy scans were performed at 25 s (for the arterial phase) and 70 s (for the venous phase) after injection. Enhancement scan parameters were as follows: tube voltage A 100 kVp; reference tube currents 230 mAs; tube voltage B 140 kVp with a tin filter; reference tube currents 178 mAs; collimation:32 × 0.6 mm; pitch: 0.55, and gantry rotation time: 0.5s.

#### Image Reconstruction and Postprocessing

The raw data were transferred to the postprocessing workstation (syngoMMWP, VE36A) to generate 120-kV equivalent mixed images (with a weighted factor of 0.5) and IM images in the venous phase. The reconstruction thickness was 1.0 mm in all cases.

### Clinical Model Development

Traditional features of patients, including clinical and semantic features, was obtained from the picture archiving and communication systems (PACS) of the hospital. Clinical features included sex (female = 0, male = 1), age (< 60 years = 0, ≥ 60 years = 1) and serum tumor markers (CEA, CA-199 and CA72-4) (negative=0, Positive=1). Two radiologists (SJY and YL with 5 and 17 years of experience in abdominal radiology, respectively) independently reviewed the venous phase CT images in PACS combined with multiple planes reconstruction (MPR) technology and recorded the semantic features using a dichotomous classification method. The radiologists knew the location of the tumor, but they were blinded to postoperative pathology. Detailed description on semantic features is provided in **Supplementary A1**. A consensus was reached through consultation in case of disagreement. The independent predictors from traditional features were identified using univariate and multivariate logistic regression for the construction of clinical model.

### Radiomics Model Development

#### Tumor Segmentation

**Figure 2:**
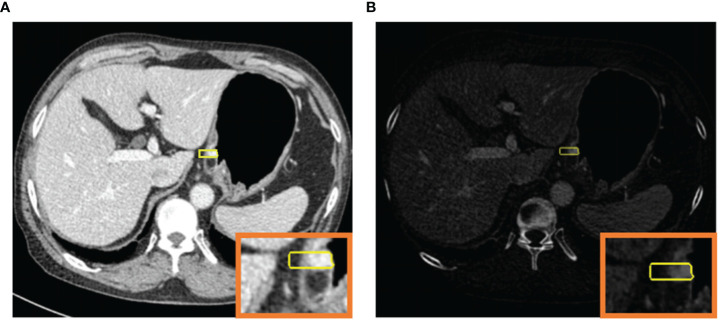
120-kV equivalent mixed image in the venous phase **(A)** and iodine map **(B)** for simultaneous ROI generation.

The mixed images and IM images were imported into a dedicated radiomics software (Radiomics, Frontier, Siemens Healthineers, Forchheim, Germany). The ROIs were delineated manually on a single layer of the mixed images by a radiologist (SJY with 5 years of experience in abdominal radiology). As **Figure 2** shows, a rectangular ROI was delineated from the mucosal to the serosal surface perpendicularly to the gastric wall, in the deepest location of tumor infiltration. The delineation slices where tumor infiltrated deepest were validated by a radiologist (YL with 17 years of experience in abdominal radiology). Differences in slice selection were resolved by consensus through negotiation. The gastric lumen, perigastric vessels, and lymph nodes were excluded. Meanwhile, the peritumoral fatty tissue within 5 mm outside the serosa was included. For ulcer type gastric cancer, the annular dike was delineated, instead of the bottom of ulcer. The ROI was automatically synchronized between the mixed image and IM with identical location, size, and shape.

#### Feature extraction and selection

The radiomics features were extracted using the Radiomics software. To ensure the reproducibility and robustness of the features, the ROIs of 50 randomly selected cases were re-drawn by same radiologist (SJY) after one month from the initial delineation. The intraclass correlation consistency analysis was used to assess the reliability of the two delineations, the features with intraclass correlation coefficient (ICC) of >0.8 were selected. The random-forest-based Boruta algorithm was used to select predictors. Boruta is a recursive feature selection algorithm by disrupt the order of feature variables and calculate the importance of feature variables to select the features with the highest importance (22). There are several methods available for feature selection based random forest algorithm. For datasets with many predictor variables, Boruta is preferable due to its higher computational efficiency (23).

#### Radiomics Modeling

The radiomics model was constructed with the R package *randomForestSRC* (24). In a random forest, multiple classification and regression trees are constructed, and the results of each tree are aggregated to make predictions for each observation. Random forest consistently provides the high prediction accuracy compared to other models (25) and is not prone to overfitting (26). A 10-fold cross-validation on the model was applied in the developing cohort to optimize the parameters (including the number of trees, the maxima depth for trees, minimum size of terminal node) of random forest classifiers. Finally, the model generalization was evaluated in the independent validation cohort.

### Establishing a Combined Model

The multivariate analysis was performed with binomial logistic regression in the statistically significant clinical factors identified by univariate analysis and multivariate analysis in clinical model development, and Rad-score. The factors with p-values < 0.05 were considered as significant predictor and used for developing combined model.

**Figure 3:**
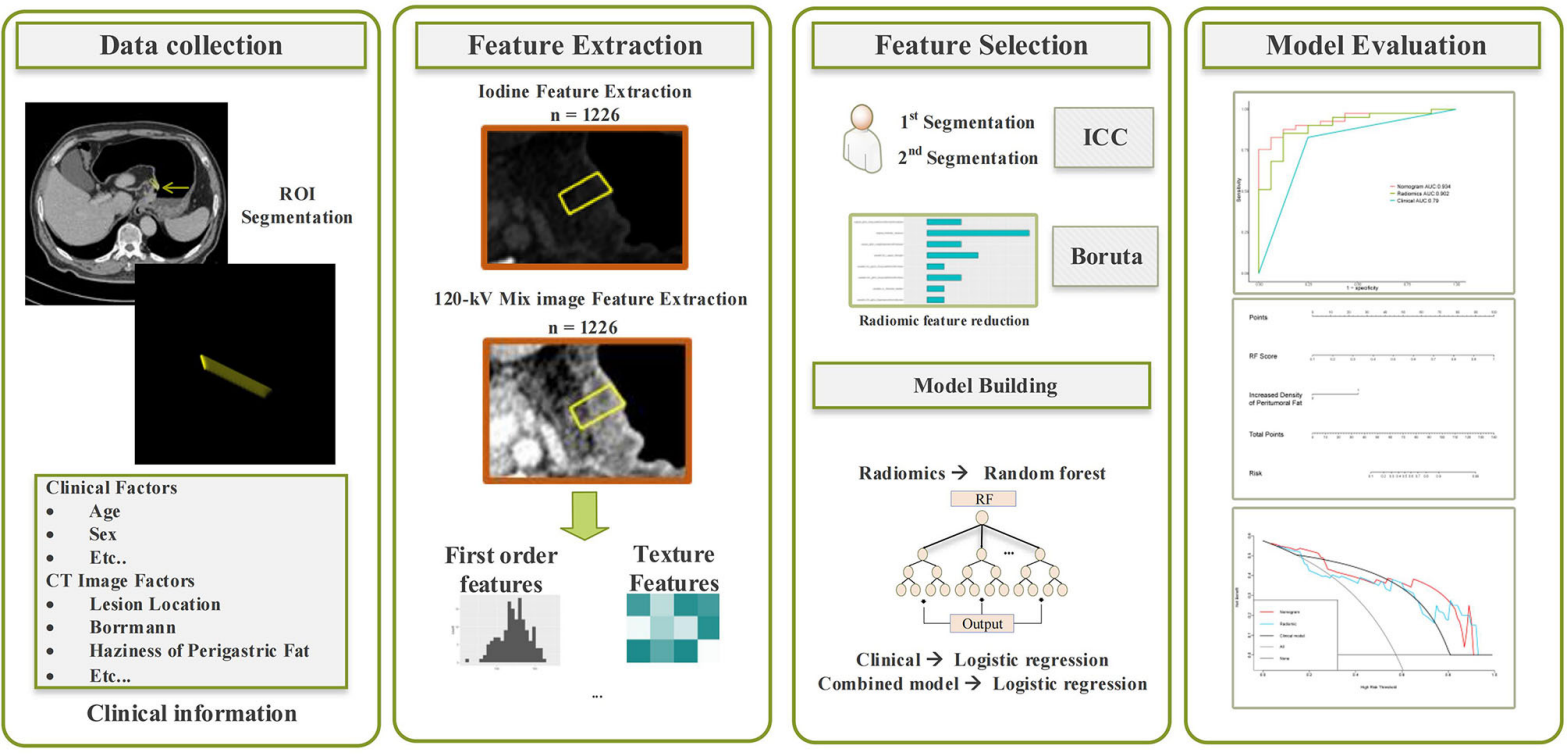
Schematic diagram of the radiomics workflow.

The radiomics workflow diagram of this study is presented in **Figure 3**.

### Statistical Methods

Kolmogorov–Smirnov test was used to test the normality of continuous variables. Independent samples *t*-test or Mann–Whitney *U* test was used for continuous variables whenever appropriate, and Fisher’s exact test or chi-square test was used for categorical variables. The diagnostic efficacies of the three models were assessed by using ROC curves, and the AUC, accuracy (ACC), sensitivity (SEN), and specificity (SPE) were calculated. Delong’s test was performed to compare the AUCs of each model and p-value < 0.0167 was considered as statistically significant for multiple comparisons according to the Bonferroni correction. Hosmer–Lemeshow test analyzed the goodness of fit for our risk prediction models and the calibration performance was evaluated by the calibration curve. The net clinical benefits were assessed by decision curve analysis (DCA).R software (version 4.0.5 http://www.Rproject.org) was used for statistical analysis and a two-sided p-value of <0.05 was used to indicate a statistically significance.

## Results

### Patients’ Characteristics

231 gastric adenocarcinoma patients (187 males, 44 females; mean age, 57.7 ± 9.9 years; age range, 27–80 years) were included in this study. Among them, 160 patients were pathologically confirmed serosal invasion-positive and 71 were negative, respectively. The training, testing, and independent validation cohort included 132, 58, and 41 patients, respectively. The traditional features of patients in the three cohorts are shown in **Table 1**.

**Table 1:** Clinical and semantic features of the patients.

Parameters	Training cohort			Testing cohort			Independent validation cohort		
	Serosa (−)	Serosa (+)	*p* value	Serosa (−)	Serosa (+)	*p* value	Serosa (−)	Serosa (+)	*p* value
	n = 38	n = 96		n = 16	n = 41		n = 17	n = 23	
**Sex**			0.236			0.313			0.631
**Female**	10 (26.3%)	15 (15.6%)		2 (12.5%)	11 (26.8%)		1 (6.25%)	4 (17.4%)	
**Male**	28 (73.7%)	81 (84.4%)		14 (87.5%)	30 (73.2%)		15 (93.8%)	19 (82.6%)	
**Age**			0.117			0.711			1
**<60 years**	13 (34.2%)	49 (51.0%)		7 (43.8%)	14 (34.1%)		7 (41.2%)	9 (39.1%)	
**≥60 years**	25 (65.8%)	47 (49.0%)		9 (56.2%)	27 (65.9%)		10 (58.8%)	14 (60.9%)	
**CEA**			0.022			0.708			0.107
**Negative**	36 (90.0%)	67 (69.8%)		14 (87.5%)	33 (80.5%)		16 (94.1%)	16 (69.6%)	
**Positive**	4 (10.0%)	29 (30.2%)		2 (12.5%)	8 (19.5%)		1 (5.88%)	7 (30.4%)	
**CA19-9**			0.079			0.170			0.123
**Negative**	37 (92.5%)	75 (78.1%)		16 (100%)	35 (85.4%)		17 (100%)	19 (82.6%)	
**Positive**	3 (7.50%)	21 (21.9%)		0 (0.00%)	6 (14.6%)		0 (0.00%)	4 (17.4%)	
**CA72-4**			0.679			0.708			1.000
**Negative**	34 (85.0%)	77 (80.2%)		14 (87.5%)	35 (85.4%)		15 (88.2%)	20 (87.0%)	
**Positive**	6 (15.0%)	19 (19.8%)		2 (12.5%)	8 (19.5%)		2 (11.8%)	3 (13.0%)	
**Localized type**	16 (42.1%)	7 (7.29%)		4 (25.0%)	2 (4.88%)		9 (52.9%)	0 (0.00%)	
**infiltrative type**	22 (57.9%)	89 (92.7%)		12 (75.0%)	39 (95.1%)		8 (47.1%)	23 (100%)	
**Thickness of the cancerous lumps**	1.30 [0.80;1.78]	1.75 [1.30;2.32]	0.001	0.94 (0.48)	1.84 (0.71)	<0.001	0.69 (0.41)	1.91 (0.47)	<0.001
**Enhancement range**			0.222			0.046			0.001
**Nontransmural**	4 (10.5%)	4 (4.17%)		4 (25.0%)	2 (4.88%)		9 (52.9%)	1 (4.35%)	
**Transmural**	34 (89.5%)	92 (95.8%)	0.073	12 (75.0%)	39 (95.1%)	<0.001	8 (47.1%)	22 (95.7%)	0.264
**Enhancement forms**			0.073			<0.001			0.264
**Homogeneous**	21 (55.3%)	35 (36.5%)		15 (93.8%)	11 (26.8%)		12 (70.6%)	11 (47.8%)	
**Heterogeneous**	17 (44.7%)	61 (63.5%)		1 (6.25%)	30 (73.2%)		5 (29.4%)	12 (52.2%)	
**Rough serosal surface**			<0.001			<0.001			<0.001
**Negative**	22 (57.9%)	8 (8.33%)		11 (68.8%)	5 (12.2%)		12 (70.6%)	1 (4.35%)	
**Positive**	16 (42.1%)	88 (91.7%)		5 (31.2%)	36 (87.8%)		5 (29.4%)	22 (95.7%)	
**Increased density of peritumoral adipose tissue**			<0.001			<0.001			<0.001
**Negative**	23 (60.5%)	8 (8.33%)		12 (75.0%)	7 (17.1%)		12 (70.6%)	2 (8.70%)	
**Positive**	15 (39.5%)	88 (91.7%)		4 (25.0%)	34 (82.9%)		5 (29.4%)	21 (91.3%)	
**Increased fat density (Rough serosal surface)**			<0.001			<0.001			<0.001
**Negative**	23 (60.5%)	8 (8.33%)		12 (75.0%)	7 (17.1%)		12 (70.6%)	2 (8.70%)	
**Positive**	15 (39.5%)	88 (91.7%)		4 (25.0%)	34 (82.9%)		5 (29.4%)	21 (91.3%)	
**Serosal nodule**			0.028			0.024			0.205
**Negative**	35 (92.1%)	70 (72.9%)		15 (93.8%)	24 (58.5%)		16 (94.1%)	17 (73.9%)	
**Positive**	3 (7.89%)	26 (27.1%)		1 (6.25%)	17 (41.5%)		1 (5.88%)	6 (26.1%)	
**Positive lymph nodes**			<0.001			0.057			<0.001
**Negative**	16 (42.1%)	11 (11.5%)		6 (37.5%)	5 (12.2%)		12 (70.6%)	2 (8.7%)	
**Abdominal and pelvic effusionPositive**	22 (57.9%)	85 (88.5%)	0.069	10 (62.5%)	36 (87.8%)	0.154	5 (29.4%)	21 (91.3%)	0.624
**Negative**	36 (94.7%)	77 (80.2%)		15 (93.8%)	31 (75.6%)		16 (94.1%)	20 (87.0%)	
**Positive**	2 (5.26%)	19 (19.8%)		1 (6.25%)	10 (24.4%)		1 (5.88%)	3 (13%)	

### Clinical Model

Univariate logistic regression analysis was performed and the two factors, i.e., rough serosal surface and increased density of peritumoral adipose tissue, were combined owing to their high collinearity with a multiple variance inflation factor (VIF) of > 10. **Supplementary Table 1** showed that CEA, the area of cancerous lumps involvement, Borrmann type, the thickness of the cancerous lumps, enhancement forms, rough serosal surface, increased density of peritumoral adipose tissue, serosal nodule, positive lymph nodes, and abdominal and pelvic effusion were significantly associated with serosal invasion (*p* < 0.05). Multivariate analysis showed that increased density of peritumoral adipose tissue was an independent predictor of serosal invasion (**Supplementary Table 2**).

### Radiomics Model

**Figure 4:**
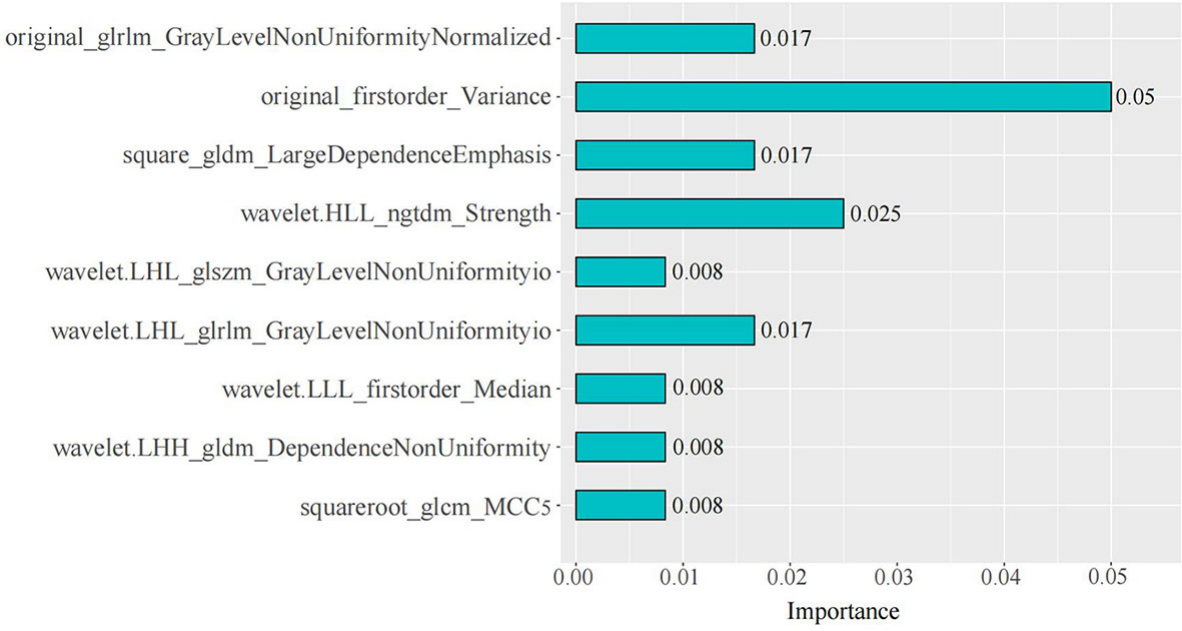
Weights of the nine radiomics features in the radiomics model.

A total of 2452 (1226 × 2) radiomics features were extracted from the mixed images and IM images for each patient, which did not include the three-dimensional information (Laplacian of Gaussian, LoG). The features of each cohort included 18 histogram features, 14 shape features, 24 gray co-occurrence matrix features, 16 gray-level run-length matrix features, 16 gray-level size zone matrix features, 5 neighboring gray tone difference matrix features, and 14 gray-level dependence matrix features. Moreover, 631 features (ICC > 0.8) were retained in the consistency analysis, and finally, 9 radiomics features with the highest mean importance were selected using Boruta for model construction. Among them, seven features were from the mixed images, including two first-order and five texture features, and two texture features were from IM. The importance of the 9 radiomics features are shown **Figure 4**. The original_firstorder_variance had the largest mean importance.

**Figure 5:**
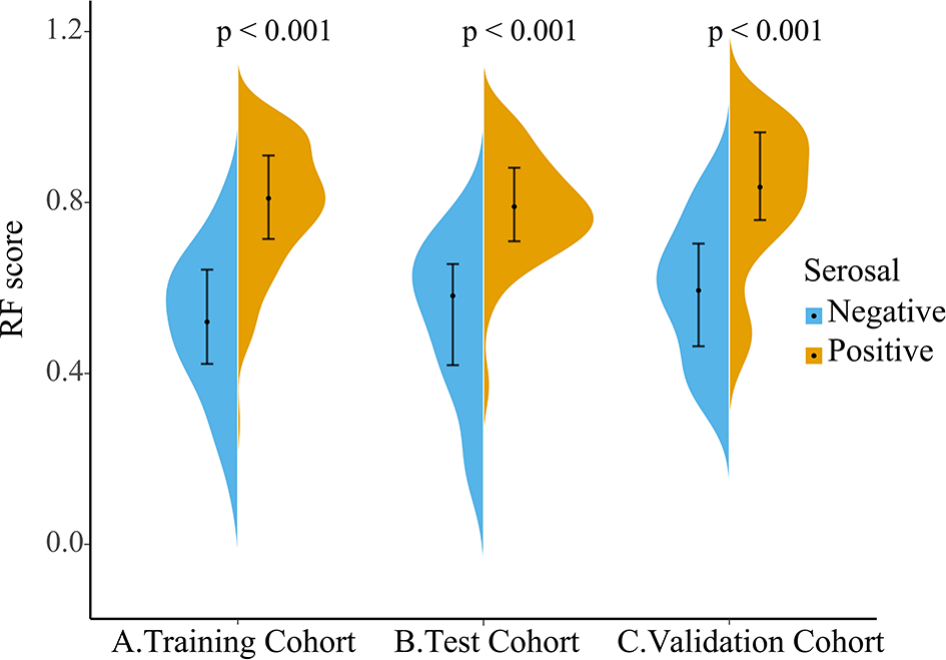
Violin plot of Rad-score distribution in the training **(A)**, testing **(B)**, and independent validation cohort **(C)**, with the outermost shape showing the density at that location. The dot on the black line is the median and the black line from the top to the end range is from the lower quartile to the upper quartile.

As the violin plot showed (**Figure 5**), The Rad-score had lower values in the serosal invasion-negative group than that in the positive group, which was statistically significantly different between the two groups in the training, testing, and independent validation cohort (all *p* < 0.001).

### Combined Model

**Figure 6:**
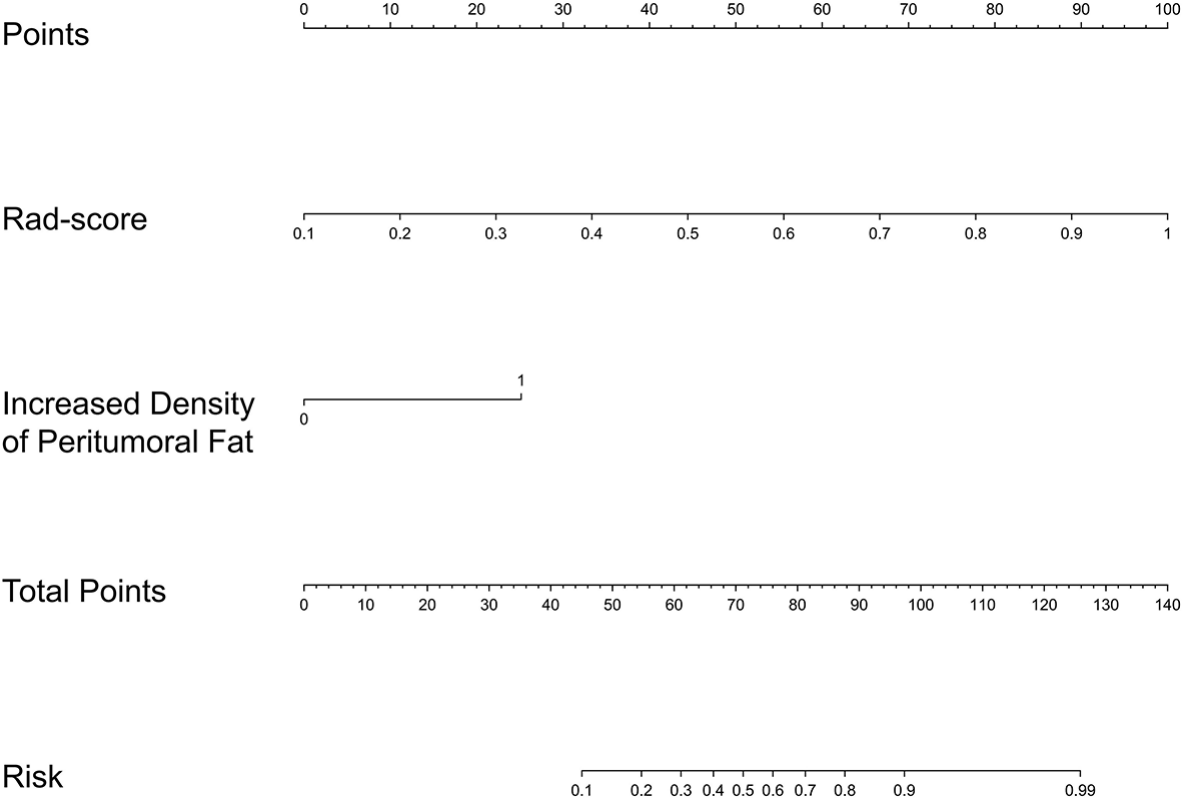
A individualized nomogram based on Rad-score and traditional feature.

**Figure 7:**
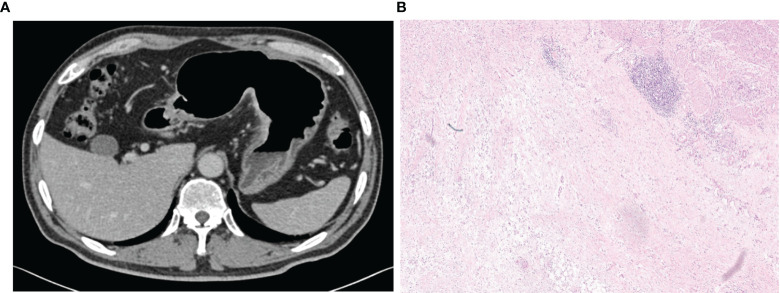
A 65-year-old male patient with increased perigastric fat density and Rad-score of 0.277,the nomogram showed a low risk of serosal invasion (risk = 0.11). **(A)** A mixed image in the portal venous phase showed the thickening of the lateral wall of the gastric lesser curvature with transmural enhancement, rough serosal surface, and increased perigastric fat density, with a positive clinical model diagnosis of serosal invasion. **(B)** Pathological images (HE, 100×) showed a large number of lymphocytes aggregated on the serosal surface with fibrous hyperplasia, and no cancer cell infiltration was observed, which was consistent with the judgement of nomogram.

As shown in **Supplementary Table 2**, the multivariate logistic regression analysis indicated the increased density of peritumoral adipose tissue and Rad-score are the independent risk factors of serosal invasion and a combined model was constructed based on the two factors. An individualized nomogram which incorporated the two predictive factors based on the combined model in training cohort was constructed to predict serosal invasion (**Figure 6**). **Figure 7** shows a typical clinically overstaged case of gastric adenocarcinoma without serosal invasion, and the nomogram showed better diagnostic performance.

### Comparison of the Three Models

Pairwise comparisons of the AUCs of the clinical model, radiomics model, and combined model were performed by the Delong’s test. As **Table 2** shows, in the training, testing, and independent validation sets, the AUC of the combined model was slightly higher than that of the radiomics model, although not statistically significant (all *p* > 0.0167). In the training cohort, the AUC of the combined model as well as the radiomics model was significantly higher than that of the clinical model (*p* < 0.01). In the testing cohort, the AUC of the combined model was significantly higher than that of the clinical model (*p* < 0.01) and the AUC of the radiomics model was slightly higher than that of the clinical model (*p* > 0.0167). And in the independent validation cohort, the AUC of the combined model and radiomics model were both slightly higher than that of the clinical model (both *p* > 0.0167).

**Table 2:** Pairwise comparisons of AUCs of the clinical model, radiomics model, and combined model.

Cohorts	AUC			*p value* (0vs1)	*p value* (0vs2)	*p value* (1vs2)
	(95%CI)					
	Clinical model (0)	Radiomics model (1)	combined model (2)			
**Training cohort**	0.76	0.9	0.93	0.002*	<0.001*	0.1191
	(0.68-0.84)	(0.85-0.95)	(0.88-0.97)			
**Testing cohort**	0.79	0.9	0.93	0.1315	0.005*	0.319
	(0.67-0.91)	(0.82-0.99)	(0.87-1.00)			
**Independent validation cohort**	0.81	0.85	0.89	0.6199	0.135	0.167
	(0.68-0.94)	(0.73-0.97)	(0.79-0.99)			

*p value＜0.0167.

In addition, pairwise comparisons of the AUCs of the radiomics model for the training, testing, and independent validation cohort were performed with Delong’s test respectively, which showed no statistical difference (all *p* > 0.0167).

**Figure 8:**
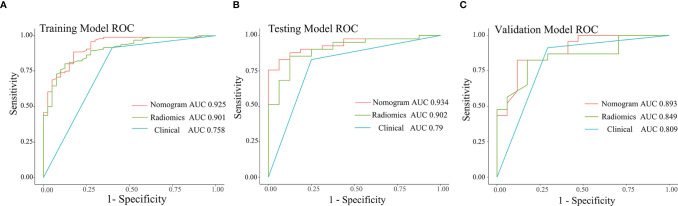
ROC curves of the three models in the training **(A)**, testing **(B)**, and independent validation **(C)** cohort.

**Figure 9:**
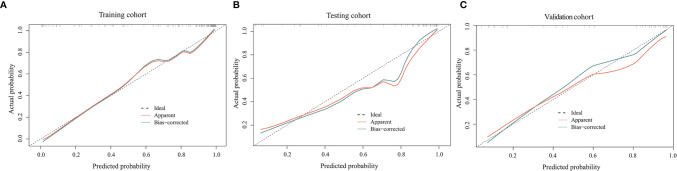
Calibration curves of the combined model in the training **(A)**, testing **(B)**, and independent validation **(C)** cohort. The horizontal axis represents the prediction probability of the traditional combined with radiomics feature model, and the vertical axis represents the actual occurrence probability, both of which demonstrated good agreement between prediction and observation of the model.

**Figure 10:**
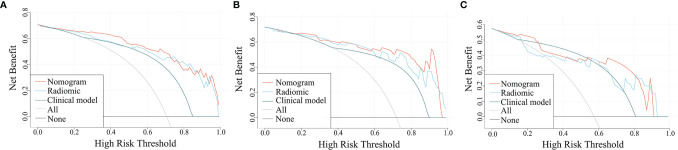
Decision curves for the three models in the training **(A)**, testing **(B)**, and independent validation **(C)** cohort. The x- and y-axes of the curve represent the threshold probability and the net benefit, respectively.

The ROC curves for the clinical model, radiomics model, and combined model are shown in **Figure 8**. **Supplementary Table 3** shows the ACC, SEN and SPE of the three models. The combined model showed the best accuracy in all three cohorts. The calibration curves (**Figure 9**) of the combined model showed great calibration performances in the training, testing and validation cohorts. There was no statistical significance performing in the three groups (*p* >0.05) through the Hosmer–Lemeshow test, which demonstrated good agreement between prediction and observation. As **Figure 10** shows, the net clinical benefit for the combined model was higher than that of the other two models when the threshold for the training, testing, and independent validation cohort was >0.09, >0.17, and >0.54, respectively.

## Discussion

Accurate preoperative determination of the serosal invasion plays a crucial role in treatment decisions of gastric cancer patients (27). In this study, we developed a radiomics model and a combined model to detect serosal invasion of gastric adenocarcinoma patients based on the mixed images and IM images derived from DECT. Both models outperformed the clinical model in the training, testing, and independent validation cohort in terms of diagnostic efficacy, and among them, the combined model performed best.

The univariate logistic regression of traditional features showed that most of the features were significantly associated with serosal invasion (*p* < 0.05), which is consistent with previous studies (28–32). Among the features, the heterogeneous enhancement of the tumor reflects heterogeneity, and numerous studies have suggested that tumor heterogeneity is related to aggressiveness (33, 34). Similarly, in this study, the weight of variance was significantly higher than the other features among the 9 radiomics features used for the radiomics model construction. As a first-order feature, variance is a measure of the spread of the distribution about the mean, which indicates the degree of data deviation. A higher value of variance may represent stronger heterogeneity of the tumor, which may suggest a higher probability of serosal invasion in cases with strong heterogeneity.

Multivariate logistic regression of the traditional features revealed that increased density of peritumoral adipose tissue was an independent predictor of serosal invasion, which was consistent with the findings of Xu Chang et al. (18) and Sun et al. (35). However, Kim et al. (12) showed that irregular or nodular changes of serosal surface were independent predictors of serosal invasion, it might be attributed to inconsistent criteria of serosal nodule among investigators. In the present study, the sign of an irregular serosal surface without a significantly localized protrusion was only classified as rough serosal surface. The rough serosal surface was almost always accompanied by increased density of peritumoral adipose tissue in cases of this study, and thus, the two signs were combined. Therefore, compared with the study of Kim et al, the frequency of increased density of peritumoral adipose tissue was higher in the patients with serosal invasion (91.7%, 88/96 vs. 75.6%, 31/41), but that of serosal nodule was lower (26/96, 27.1% vs. 75.6%, 31/41), which suggested that there were differences in the interpretation of subjective signs by different observers. Thus, quantitative parameters are required for more objective determination for the serosal status.

Cutting-edge technologies such as IM derived from DECT and radiomics enable the quantitative assessment of lesion features (14–17). In this study, we established a radiomics model and a combined model based on DECT to detect serosal invasion. Unlike previous studies, we explored a new segmentation method, which is more timesaving than the whole tumor segmentation, avoiding errors due to the inaccurate determination of tumor boundaries. Meanwhile, peritumoral adipose tissue was included in the ROI, which helped to assess tumor aggressiveness more comprehensively. The results showed that the diagnostic performance of the combined model in the present study was superior to that of the combined model based on conventional enhanced CT (AUCs of testing set, 0.93 vs. 0.812) (16), and was comparable with that of the combined model based on DECT from Wang L et al.’s study (19) (AUCs of testing set, 0.93 vs. 0.914). The whole tumor segmentation was used in both two previous studies.

There were several limitations in this study. Firstly, this was a single-center study with relatively small sample size. Although an independent validation cohort was established, the results obtained herein still need to be validated based on multicenter data. Secondly, the width of ROI was not limited in spite of the relatively fixed delineation depth. And thus, the radiomics features with ICC of >0.8 only accounted for 25.7% (631/2452). Therefore, this proposed delineation method needs to be further standardized. In addition, 13 patients were excluded from the study due to the lack of peritumoral adipose tissue; it can be seen that the results of this study may not be applicable to certain patients with cachexia.

This study showed that the radiomics model and combined model, which were constructed based on tumoral and peritumoral radiomics features based on DECT, have good diagnostic efficacy and could be a useful tool to determine the serosal invasion for gastric adenocarcinoma.

## Data Availability Statement

The raw data supporting the conclusions of this article will be made available by the authors, without undue reservation.

## Ethics Statement

The studies involving human participants were reviewed and approved by the Ethics Committee of The Fourth Hospital of Hebei Medical University. Written informed consent for participation was not required for this study in accordance with the national legislation and the institutional requirements.

## Author Contributions

LY and JS designed the study. JS and YL wrote the initial draft of the manuscript. XY performed the statistical analysis. ML, JL, and XW collected CT images and clinical data. LY and GS performed manuscript approval and modification. All authors contributed to the article and approved the submitted version.

## Conflict of Interest

Author XY was employed by Siemens Healthineers Ltd.

The remaining authors declare that the research was conducted in the absence of any commercial or financial relationships that could be construed as a potential conflict of interest.

## Publisher’s Note

All claims expressed in this article are solely those of the authors and do not necessarily represent those of their affiliated organizations, or those of the publisher, the editors and the reviewers. Any product that may be evaluated in this article, or claim that may be made by its manufacturer, is not guaranteed or endorsed by the publisher.
