# Supplementary material for: Diagnosis of Serosal Invasion in Gastric Adenocarcinoma by Dual-Energy CT Radiomics: Focusing on Localized Gastric Wall and Peritumoral Radiomics Features
Source: Front Oncol. 2022 Mar 21;12:848425. doi: 10.3389/fonc.2022.848425 (PMC8977467; doi:10.3389/fonc.2022.848425)
Supplement: Supplementary file 1 [file DataSheet_1.docx]

Supplementary Material

# Supplementary A1: The semantic characteristic

1. **The area of cancerous lumps involvement**: The stomach was divided into 5 areas: cardia, fundus, body, gastric angle, and antrum. Cancerous lumps involving 1 area = 0 and those involving ≥2 areas = 1.
2. **Borrmann type**: Including localized type (type I: nodular type and type II: ulcer-localized type) and infiltrative type (type III: ulcer-infiltrative type and type IV: diffuse-infiltrative type). Localized type = 0 and infiltrative type = 1.
3. **The thickness of the cancerous lumps**: The maximum-sized layer of the cancerous lump was selected for the measurement of the thickness of tumor, which was perpendicular to the stomach wall adopting multiple planes reconstruction (MPR) technology. The thickness of the annular dikes of the ulcer-type tumor was measured. Sites, which were adhesive to the perigastric lymph nodes, should be bypassed, and the perigastric adipose tissue with tumor infiltration was excluded (< mean value = 0 and ≥ mean value = 1).
4. **Enhancement range**: Nontransmural enhancement = 0 and transmural enhancement = 1.
5. **Enhancement forms**: The difference between the maximum and the minimum computed tomograhy (CT) attenuation value of the tumor in the venous phase of ≥10 HU was defined as heterogeneous enhancement, whereas <10 HU was defined as homogeneous enhancement. Homogeneous enhancement = 0 and heterogeneous enhancement = 1.
6. **Rough serosal surface**: Manifesting as the area of serosal cords exceeding 1/3 of the lesion area. Negative = 0 and Positive = 1.
7. **Increased density of peritumoral adipose tissue**: Refers to CT attenuation value of the peritumoral adipose tissue 10 HU higher than that of the normal perigastric adipose tissue. Negative = 0 and Positive = 1.
8. **serosal nodule**: Manifesting as a localized nodular protrusion on the serosal surface. Negative = 0 and Positive = 1. of
9. **Positive lymph nodes**:CT diagnostic criteria of positive lymph nodes were as follows: round or oval; enhanced; disppearment of the hilus of the lymph nodes; short axis diameters of ≥0.7 cm; small and clustered, and sentinel lymph nodes. Negative = 0 and Positive = 1.
10. **Abdominal and pelvic effusion**: Negative = 0 and Positive = 1.

# Supplementary Tables

**Table 1: Correlations between serosa positive and traditional characteristics**

| **Variables** | ***β*** | **OR(95%CI)** | ***p* value** |
| --- | --- | --- | --- |
| **Sex** | 0.588 | 1.800(0.713-4.414) | 0.202 |
| **Age** | 0.773 | 0.462(0.208-0.986) | 0.05 |
| **CEA** | 1.360 | 3.896(1.397-13.897) | 0.017 |
| **CA19-9** | 1.239 | 3.453(1.100-15.28) | 0.056 |
| **CA72-4** | 0.335 | 1.398 (0.537-4.116) | 0.512 |
| **The area of cancerous lumps involvement:** | 2.03 | 7.615(2.779-26.927) | ＜0.001 |
|  |  |  |  |
| **Borrmann type** | 2.24 | 9.398(3.613-26.895) | ＜0.001 |
| **The thickness of the cancerous lumps:** | 1.021 | 2.775(1.594-5.247) | ＜0.001 |
| **Enhancement range** | 0.938 | 2.556(0.577-11.338) | 0.201 |
| **Enhancement forms** | 0.756 | 2.130(1.011-4.550) | 0.048 |
| **Rough serosal surface** | 2.7 | 14.882(5.938-40.933) | ＜0.001 |
| **Increased density of peritumoral adipose tissue** | 2.803 | 16.500(6.568-45.598) | ＜0.001 |
| **Increased density of peritumoral adipose tissue（Rough serous surface)** | 2.803 | 16.500(6.568-45.598) | ＜0.001 |
| **Serous nodule** | 1.522 | 4.581(1.485-20.101) | 0.018 |
| **Positive lymph node** | 1.742 | 5.711(2.386-14.249) | ＜0.001 |
| **Abdominal and pelvic fluid** | 1.545 | 4.688(1.271-30.389) | 0.045 |

**Table 2: Variables and coefficients of combined and clinical models**

| **Variables** | **Clinical model** | | | **Combined model** | | |
| --- | --- | --- | --- | --- | --- | --- |
|  | ***β*** | **OR (95% CI)** | ***P* value** | ***β*** | **OR (95%CI)** | ***p* value** |
| **Intercept** | -1.634 |  |  | -6.947 |  |  |
| **Increased density of peritumoral adipose tissue** | 2.05 | 7.767 (2.42-28.242) | ＜0.001 | 1.925 | 6.857  （1.660-33.014） | 0.010 |
| **Rad-score** | **-** | **-** | **-** | 10.022 | 22517.547 （429.152-3043145.434） | <0.001 |

**Table 3: The diagnostic efficacy of the clinical model, radiomics model, and combined model in the training, testing, and independent validation set**

| **Models** | **Training cohort** | | | **Testing cohort** | | | **Independent validation cohort** | | |
| --- | --- | --- | --- | --- | --- | --- | --- | --- | --- |
|  | **ACC** | **SEN** | **SPE** | **ACC** | **SEN** | **SPE** | **ACC** | **SEN** | **SPE** |
|  | **（95%CI）** | **（95%CI）** | **（95%CI）** | **（95%CI）** | **（95%CI）** | **（95%CI）** | **（95%CI）** | **（95%CI）** | **（95%CI）** |
| **Clinical model** | 0.82  (0.76-0.88) | 0.92  (0.85-0.97) | 0.60  (0.43-0.75) | 0.81  (0.70-0.89) | 0.83  (0.71-0.93) | 0.75  (0.50-0.94) | 0.83  (0.73-0.93) | 0.91  (0.78-1.00) | 0.71  (0.47-0.88) |
|  |  |  |  |  |  |  |  |  |  |
| **Rradiomics model** | 0.84  (0.75-0.93) | 0.85  (0.73-0.95) | 0.81  (0.63-1.00) | 0.82(0.76-0.88) | 0.80  (0.72-0.88) | 0.88  (0.78-0.98) | 0.80  (0.68-0.90) | 0.87  (0.74-1.00) | 0.71  (0.47-0.88) |
|  |  |  |  |  |  |  |  |  |  |
| Combined  model | 0.88  (0.79-0.95) | 0.88(0.78-0.98) | 0.88  (0.69-1.00) | 0.87  (0.81-0.92) | 0.89  (0.82-0.95) | 0.83  (0.70-0.93) | 0.83  (0.70-0.93) | 0.83  (0.65-0.96) | 0.82  (0.65-1.00) |

*ACC*, accuracy; *SEN*, sensitivity; *SPE*, Specificity
